# Low cholesteryl ester transfer protein and phospholipid transfer protein activities are the factors making tree shrew and beijing duck resistant to atherosclerosis

**DOI:** 10.1186/1476-511X-9-114

**Published:** 2010-10-12

**Authors:** Hui-rong Liu, Gang Wu, Bing Zhou, Bao-sheng Chen

**Affiliations:** 1 College of Life Sciences, Inner Mongolia Agricultural University, Hohhot 010018, China; 2 National Laboratory of Medical Molecular Biology, Institute of Basic Medical Sciences, Chinese Academy of Medical Sciences, Peking Union Medical College, Beijing 100005, China; 3 Beijing Center for ADR Monitoring, Beijing 100011, China

## Abstract

**Background:**

Tree shrew and beijing duck are regarded as animal models resistant to atherosclerosis (AS). This study was carried out to discover the potential mechanism.

**Methods:**

Blood samples were collected from healthy men and male animals. Plasma lipid profile and activities of cholesteryl ester transfer protein (CETP) and phospholipid transfer protein (PLTP) were measured, compared and analyzed in human, tree shrew, and Beijing duck.

**Results:**

The results showed that there were species differences on plasma lipid profile and activities of CETP and PLTP in the three species. Compared with human, tree shrew and beijing duck had higher high density lipoprotein cholesterol (HDL-C)/total cholesterol (TC) and HDL-C/low density lipoprotein cholesterol (LDL-C) ratios, but lower CETP and PLTP activities. In the three species, CETP and PLTP activities were negatively related with the ratio of HDL-C/LDL-C.

**Conclusions:**

The present study suggested that low plasma CETP and PLTP activities may lead to a high HDL-C/LDL-C ratio and a high resistance to AS finally in tree shrew and beijing duck. Moreover, low PLTP activity may also make the animals resistant to AS by the relative high vitamin E content of apoB-containing lipoproteins and high anti-inflammatory and antioxidative properties of HDL particles. A detailed study in the future is recommended.

## Background

Cholesteryl ester transfer protein (CETP) and phospholipid transfer protein (PLTP) are two important factors to transfer lipids in lipoprotein metabolism. CETP transfers cholesteryl ester from high density lipoprotein (HDL) to lipoproteins of lower density, partly in exchange for triglycerides. PLTP promotes the transfer of phospholipids and free cholesterol between lipoproteins [1,2]. It is reported that CETP and PLTP activities are closely related to atherosclerosis (AS) [1,2].

Some vertebrate species can be defined as two distinct groups with low or high atherosclerosis susceptibility [3]. Cat, dog, mouse and rat belong to the 'resistant' group, while chicken, pig, rabbit and man belong to the 'susceptible' group. It is showed that plasma lipids transfer activities are significantly different between the two groups, which exert different effects on the atherogenicity of the plasma lipoprotein profile and therefore make the species resistant or susceptible to atherosclerosis [4].

Tree shrew and beijing duck are regarded as animal models resistant to AS [5,6]. It is documented that plasma HDL still remains a relative high level in these animals and no typical atherosclerotic plaques are found in the artery wall on a high cholesterol diet. These data point out a high resistance of tree shrew and beijing duck to AS, however, the potential mechanism still remains unknown.

In this study, we measured and compared plasma lipid profile and activities of CETP and PLTP in human, tree shrew and beijing duck, which might provide some helpful insights on the anti-atherosclerotic mechanism in the two species.

## Methods

### Blood samples

Blood samples from 20-30-year old normolipidemic men were provided by Central Blood Bank of Peking Union Medical College Hospital, and blood samples from 6-month old male tree shrews were provided by Kunming Institute of Zoology, Chinese Academy of Science. Blood samples from 4-month old male beijing ducks were generous gifts of Prof. Suisheng Hou [Institute of Animal Science (IAS), Chinese Academy of Agricultural Sciences (CAAS)]. The blood samples were collected into EDTA-containing glass tubes by veno-puncture. Plasma was separated by a 5-min centrifugation at 3000 g at 4°C and was immediately stored at -70°C for further analysis. The experiments were performed in accordance with the guidelines of the National Institutes of Health (Bethesda, MD, USA) and Peking Union Medical College for the humane treatment of laboratory animals. All efforts were made to minimize the animals' suffering.

### Plasma lipid assays

Total plasma cholesterol (TC) was measured using CHOD-PAP method (cholesterol oxidase peroxidase-phenol 4 amino phenazon), and plasma triglyceride (TG) was measured using the GPO-PAP method (Glycerol phosphate oxidase-phenol 4 amino phenazon) (Zhongshengbeikong Biotech Co., Ltd, Beijing, China). High density lipoprotein cholesterol (HDL-C) and low density lipoprotein cholesterol (LDL-C) were determined by homogeneous method [synthetic polymer/detergent HDL-C assay (SPD) method for HDL-C and surfactant LDL-C assay (SUR) method for LDL-C] (Daiichi Pure Chemicals Co. LTD., Japan).

### Plasma CETP and PLTP activity

Plasma CETP activity was measured using an assay kit following the manufacturer's instruction (BioVision, Mountain View, CA). Briefly, 3 μl of plasma sample (as the source of CETP) was added to the reaction mixture containing a fluorescent self-quenched neutral lipid as the donor molecule and an acceptor molecule. A CETP-mediated transfer of the fluorescent neutral lipid to the acceptor molecule resulted in an increase in fluorescence, which was read in a fluorescence plate reader at excitation 465 nm and emission 535 nm. CETP activity was expressed as pico-mole of neutral lipid transferred per microlitre plasma per hour. Samples were run in triplicate. All CETP analyses were conducted in the same day to decrease variability. Plasma PLTP activity was determined using a PLTP activity kit (Bio Vision). The protocol for the PLTP activity kit was similar to that for the CETP activity kit.

### Statistical analysis

Data were analyzed using SPSS for Windows XP. All descriptive data collected were expressed as mean ± standard deviation (SD) and the Student '*t*' test was used for analysis. Values of P < 0.05 were regarded as statistically significant.

## Results

### Plasma Lipid concentrations

Lipid concentrations were measured in total plasma of human, tree shrew and beijing duck (Table 1). In the three species, levels of TC, TG, and HDL-C were highest in beijing duck, whereas levels of LDL-C were highest in human. Plasma HDL-C/TC and HDL-C/LDL-C ratios were higher in tree shrew and Beijing duck than in human (Table 2).

**Table 1:** Plasma lipid concentrations in human, beijing duck and tree shrew

Species	n	TC(mg/dl)	TG(mg/dl)	HDL-C(mg/dl)	LDL-C(mg/dl)
Human	9	137.75 ± 30.26	63.75 ± 22.59	47.38 ± 9.05	78.25 ± 24.70
Beijing duck	12	166.27 ± 30.38	268.82 ± 54.57	83.45 ± 15.96	42.91 ± 12.20
Tree shrew	9	66.44 ± 9.02	88.22 ± 16.39	29.11 ± 5.16	11.56 ± 3.00

The values are mean ± S.D.

**Table 2:** Activities of plasma CETP and PLTP and ratios of HDL-C/TC and HDL-C/LDL-C in human, beijing duck and tree shrew.

		CETP activity	PLTP activity		
Species	n	(pmol/μl/h)	(pmol/μl/h)	HDL-C/TC	HDL-C/LDL-C
Human	9	59.7 ± 10.5	127.1 ± 18.7	0.33 ± 0.07	0.60 ± 0.24
Beijing duck	12	26.1 ± 5.6	80.8 ± 7.8	0.50 ± 0.03	1.99 ± 0.37
Tree shrew	9	23.8 ± 4.4	63.2 ± 12.7	0.44 ± 0.05	2.70 ± 1.01

The values are mean ± S.D. of three independent experiments.

### Plasma cholesteryl ester transfer activity

**Figure 1:**
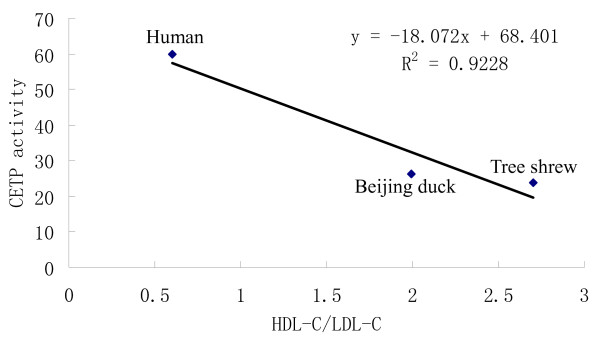
**Relationship between the CETP activity and the ratio of HDL-C/LDL-C**.

As described in section 2, cholesteryl ester transfer activity among the three species was evaluated by using CETP activity assay kit (Table 2). Cholestryl ester transfer rates could be measured in all the studied species. Plasma CETP activity in human was highest among the three species, about 59.7 ± 10.5 pmol/μl/h. Plasma CETP activities in beijing duck and tree shrew were greatly lower than that in human, respectively 26.1 ± 5.6 and 23.8 ± 4.4 pmol/μl/h. In the three species, CETP activity was negatively related with the ratio of HDL-C/LDL-C (*r *= -0.96063) (Fig. 1).

### Plasma phospholipid transfer activity

**Figure 2:**
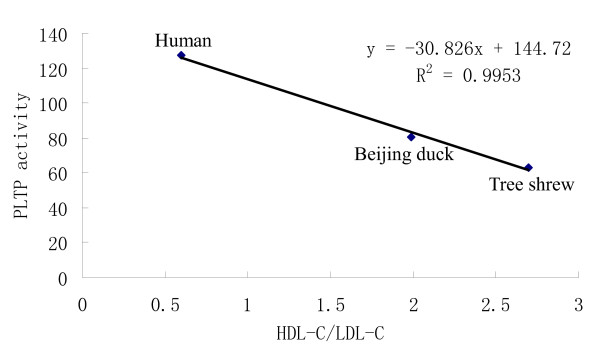
**Relationship between the PLTP activity and the ratio of HDL-C/LDL-C**.

Plasma PLTP activity was determined by using PLTP activity assay kit (Table 2). Plasma PLTP activity in human was about 127.1 ± 18.7 pmol/μl/h, dramatically higher than that in beijing duck and tree shrew (respectively 80.8 ± 7.8 and 63.2 ± 12.7 pmol/μl/h). In the three species, PLTP activity was also negatively related with the ratio of HDL-C/LDL-C (*r *= -0.99763) (Fig. 2).

## Discussion

In our study, compared with human, tree shrew and beijing duck had high HDL-C/TC and extremely high HDL-C/LDL-C ratios. In human, the HDL-C level was lower than the LDL-C level. The HDL-C/LDL-C ratio was about 0.60. However, in tree shrew and beijing duck, the HDL-C level was greatly higher than the LDL-C level. The HDL-C/LDL-C ratio even reached to 2.7 in tree shrew and 1.99 in beijing duck. It's well known that HDL-C is positively and LDL-C is negatively related with cardiovascular disease [7-9]. When too much LDL-C circulates in the blood, it can pass from the blood into the arterial wall where it may be oxidised and engulfed by macrophages forming foam cells. Thereafter, a complex interplay of cell necrosis, smooth muscle recruitment and collagen deposition leads to the development of atherosclerotic plaques. However, HDL is involved in the reverse transport of cholesterol from the peripheral tissues to the liver, thereby reducing the uptake of cholesterol by macrophages and providing a protective effect against atherosclerosis. It also has anti-inflammatory, antioxidant, or other effects on the arterial wall. Therefore, to reduce the risk of cardiovascular disease, many strategies have been adopted to decrease the circulating LDL-C level and increase HDL-C level. Our study demonstrated that tree shrew and beijing duck had very high HDL-C level but low LDL-C level, which might be the reason why the two species were resistant to AS.

It is reported that mean CETP activity is markedly lower in the 'resistant' group than in the 'susceptible' group, but PLTP activity is higher in the 'resistant' group than in the 'susceptible' group [4]. In accordance with the previous findings, in our study, as the species resistant to AS, tree shrew and beijing duck had dramatically lower plasma CETP activity than human, the species susceptible to AS. However, PLTP activity in the two species was also greatly lower than that in human, which was significantly different from the previous report [4]. This may be because of the species differences. Furthermore, in the three species, CETP and PLTP activities were negatively related with the ratio of HDL-C/LDL-C, not as reported that CETP activity was inversely correlated with HDL-C/TC ratio [10]. CETP and PLTP are two important factors to transfer lipids in lipoprotein metabolism. CETP transfers cholesteryl ester from HDL to lipoproteins of lower density, partly in exchange for triglycerides. PLTP promotes the transfer of phospholipids and free cholesterol between lipoproteins [1,2]. Much research shows that the deficiency or decrease of CETP activity can lead to an increase in HDL-C, a decrease in LDL-C, and a lower incidence of stroke and coronary heart disease (CHD) [11-13]. However, the increase of CETP activity decreases the HDL-C level and increases the LDL-C level and atherosclerosis [14-16]. Although there are some contrary results too [17,18], most of the studies seem to suggest that the partial inhibition of CETP appear to provide a reduction in atherosclerosis and CHD risk. Numerous in vivo studies show that an increase in plasma PLTP activity is associated with decreased HDL-C level, increased hepatic VLDL secretion and increased atherosclerotic lesion development [19-25]. In addition to its effect on HDL-C level and VLDL secretion, PLTP can also influence the atherogenicity of plasma lipoproteins by decreasing the vitamin E content of apoB-containing lipoproteins and affecting the anti-inflammatory and antioxidative properties of HDL particles, which results in increased susceptibility to AS [23,24]. On the contrary, PLTP deficiency results in markedly decreased atherosclerosis by a decrease in the production and levels of apoB-containing lipoprotein, an increase in their vitamin E content, and a decrease in their susceptibility to oxidation [23,26]. It is demonstrated that pronounced higher CETP and PLTP activities can lead to a marked decrease in HDL-C levels that is due to hypercatabolism [1,27-29] and an increase in LDL-C levels that is due to an increase in transfer of CE from HDL to LDL and production of apoB-containing lipoprotein [1,26,30,31]. In tree shrew and beijing duck, low CETP and PLTP activities might cause a low catabolism of HDL-C and a decrease in production of apoB-containing lipoprotein, which led to a relative high plasma level of HDL-C and low level of LDL-C and at last a high resistance to AS. Moreover, low PLTP activity may also make the animals resistant to AS by the relative high vitamin E content of apoB-containing lipoproteins and high anti-inflammatory and antioxidative properties of HDL particles. Of course, these details need to be studied further in the future.

Atherosclerotic cardiovascular disease (CVD) is the leading cause of morbidity and mortality in the industrialized world. The blood concentrations of the lipoprotein cholesterol fractions are closely related to the incidence of CVD. Many attempts have been made to control the blood lipoprotein cholesterol concentrations by regulating the activities of those enzymes involved in the lipoprotein metabolism. Therefore, the completion of our work may provide some helpful insights on the clinical treatment of human AS in the future.

## Conclusions

The present study suggested that low plasma CETP and PLTP activities may lead to a high HDL-C/LDL-C ratio and a high resistance to AS finally in tree shrew and beijing duck. Moreover, low PLTP activity may also make the animals resistant to AS by the relative high vitamin E content of apoB-containing lipoproteins and high anti-inflammatory and antioxidative properties of HDL particles. A detailed study in the future is recommended.

## Competing interests

The authors declare that they have no competing interests.

## Authors' contributions

HRL was the main investigator, conceived the study, carried out experiments, collected the data, and drafted the manuscript. BSC supervised the research and contributed to all aspects of the study. GW and BZ was advisor of the study and helped in analysis and interpretation. All authors read and approved the final manuscript.
